# Constructing indices representing supportiveness of the physical environment for walking using the Rasch measurement model

**DOI:** 10.1186/1479-5868-3-44

**Published:** 2006-12-16

**Authors:** Gavin R McCormack, Louise C Mâsse, Max Bulsara, Terri J Pikora, Billie Giles-Corti

**Affiliations:** 1 School of Population Health, The University of Western Australia, 35 Stirling Highway, Crawley, Western Australia, 6009, Australia; 2 Department of Pediatrics, The University of British Columbia, Centre for Community Child Health Research, 4480 Oak St., L408, Vancouver, British Columbia, V5H 3V4, Canada

## Abstract

**Background:**

The objectives of this study were to use the Rasch model to 1) assess the psychometric properties of a physical environmental audit instrument and 2) to develop indices of interrelated environmental attributes that summarize environmental supportiveness for walking.

**Methods:**

A set of items were derived representing two conceptual physical environmental constructs: 1) functional/safety, and; 2) aesthetics. *Ad hoc *criteria based on point-biserial and Rasch-based fit statistics were used to examine the construct validity and internal reliability of the two constructs.

**Results:**

The Rasch-based fit statistics assisted in identifying 12 items that belonged to the functional/safety construct and 4 items that belonged to the aesthetic construct. The reliability of the two constructs were low to moderate (functional/safety r_β _= 0.19 and aesthetics r_β _= 0.35).

**Conclusion:**

Given the vast number of built environmental attributes, a means of developing summary indices is essential. Future studies should assess the reliability and validity of indices that summarize physical environmental characteristics conducive to walking before testing them in predictive models of physical activity. More research examining procedures for measuring the built environment and techniques for analyzing environmental data are needed to guide future research in this area.

## Background

In the past decade, understanding the impact of the physical environment on physical activity has become a topic of increasing interest. Recent reviews have highlighted characteristics of the physical environment associated with physical activity behaviors [1-3]. To date measures of the physical environment have generally included self-reported perceptions, objectively measured audit data or Geographical Information Systems (GIS) data[1,2].

Environmental attributes of the physical environment do not necessarily affect physical activity behavior in isolation; however, aggregates of these environmental attributes (i.e., sprawl index, neighborhood walkability index, functionality index, safety index, aesthetics index) have been developed and found to predict physical activity behavior [4-6]. A recent review of audit instruments used to assess the supportiveness of the environment for physical activity found that few studies had evaluated the psychometric properties of their instruments[7]. As the predictive validity of these indices is affected by the psychometric properties of these instruments and the methodology employed to aggregate items, there is a need to use more stringent methodologies to develop valid and reliable environmental audit instruments.

The Systematic Pedestrian and Cycling Environmental Scan (SPACES) instrument[8] was developed to measure attributes of the physical environment that are theoretically associated with walking and cycling in neighborhoods. It was designed to be used by trained observers to collect environmental data at a segment level (i.e., a segment is defined as the section of road between two intersections) and has been applied in studies involving urban neighborhoods[6,9]. The content representation of the instrument was assessed[10] and the instrument has been shown to be reliable[8]. However to date, other important statistical attributes of the SPACES's items and procedures for aggregating the items to form summary environmental indices have not been examined. Item response models, including the Rasch model provides an integrated approach to examine properties of items and scale that are not available with classical test theory procedures or with factor analysis. Item response modeling allows for the following to be assessed: 1) dimensionality of the scale; 2) location of items on the continuum measured by the construct; 3) evaluation of content representation or the extent to which the construct measured is adequately covered; 4) reliability and standard error of measurement of the scale across the construct; and 5) functioning of the response format or scoring model for each item [11,12].

Item response models, including the Rasch model, can be used to assess the psychometric properties of the items and scales. The Rasch model differs from other types of item response models in that only one-parameter is estimated (i.e., the "difficulty" parameters). The difficulty parameter represents the amount of an attribute an item demands of the underlying construct being measured [13]. Applying this to the theme of this paper – environmental settings that are more supportive for walking are more likely to have rare or less common environmental attributes or features (i.e., a higher difficulty parameter) compared with environmental settings that are less supportive for walking (i.e., a lower difficulty parameter). The Rasch model satisfies the requirements for fundamental measurement meaning that properties of the measure is invariant across both people and items, in other words the individual's trait or construct can be calculated independently of the difficulty of items, and vice versa[11,14] Furthermore, the Rasch model provides a method for constructing linear interval level scales from ordinal level data[15], thus representing quantitatively the underlying construct.

Therefore, the main purpose of this study was to build upon research undertaken by Pikora and colleagues[8,10] and to examine how environmental audit variables are interrelated. To do this the first objective was to examine the statistical properties, including construct validity and reliability, of environmental variables derived from SPACES and other external environmental data sources using the Rasch model. The second objective was to derive a set of environmental variables for inclusion in environmental indices representing unidimensional measures of environmental supportiveness for walking.

## Methods

### Sample

During February to April 2000, objective environmental data were collected from 12,925 segments within a 408 km^2 ^area of Perth, Western Australia[8]. The data collection was part of a larger study known as the Study of Environmental and Individual Determinants of Physical Activity 2 – (SEID 2). Segment data were collected from 1803 neighborhoods. A neighborhood was defined as the area within a 400 meter linear distance from a respondent's home. The respondents participated in an earlier cross-sectional survey (SEID 1)[16]. The sampling frameworks for SEID 1 and 2 are described more fully elsewhere[8,17]. The current study included only those segments with complete environmental data (n = 10,169 segments). From this dataset, two random samples, an exploratory sample (n = 5051) and a validation sample (n = 5118), were generated using SPSS 12.

### Environmental constructs

Pikora et al's[10] conceptual framework posits four environmental constructs: functional, safety; aesthetics; and destinations. However, based on recent evidence[1,2,18,19], the current study included two constructs 1) functional/safety, and 2) aesthetics. Aspects of safety were considered to be related to the functional environment because it included physical attributes such as the presence of crossing devices, street lights and street surveillance (i.e., physical environment conducive for observing the street from the household). In addition, the framework posits that traffic attributes can contribute to the functionality and safety of the built environment [10]. Destinations was included as a single item in the functional/safety construct as it captured whether a destination was present in the segment. The aesthetics construct included items that reflected the attractiveness or visual appeal of the streetscape.

### Environmental items reflecting constructs

The SPACES is a 35-item instrument used in SEID 2 to collect segment level data on physical environmental attributes hypothesized to be associated with walking and cycling[8,10]. In addition to field observations, data were also collected from external sources using Geographical Information Systems (GIS). The data collected and their sources have been described elsewhere[8]. Only those items relevant to the constructs were included in the current study.

Composite items including data from both SPACES and the other above mentioned sources were developed because responses to some items were dependent on responses to other items. For example, the presence of *traffic control devices *are generally present on major roads, rather than in cul-de-sacs. Hence, a variable which represented a combination of both road type and presence of traffic control devices was derived. Deriving composite items reduce the chance of violating the Rasch model assumption of local independence[11,20]. Eight derived composite items included *traffic control devices *(i.e., road type and traffic control device),*crossing devices *(i.e., road type and crossing devices),*crossing aids *(i.e., road type and crossing aids),*path/road condition *(i.e., road condition and path condition),*slope of path/road *(i.e., slope of path and slope of road),*path location from road *(i.e., presence of path and path location),*views *(combination of view types), and *trees *(presence and number of trees). Table 1 list functional/safety (herein referred to as 'functional') and aesthetic items and their category coding used in this study.

**Table 1:** Variable descriptions and category response scores

**Variables**	
**Functional construct**	

Traffic control devices^4^	0 (major road/no device); 1 (major road w/device); 2 (minor road/no device); 3 (minor road w/device); 4 (cul-de-sac with/without device)
Crossing devices ^4^	0 (major road/no device); 1 (major road w/device); 2 (minor road/no device); 3 (minor road w/device); 4 (cul-de-sac with/without device)
Crossing aids ^4^	0 (major road/no aid); 1 (major road w/aid); 2 (minor road/no aid); 3 (minor road w/aid); 4 (cul-de-sac with/without aid)
Road width^4^	0 (4+ lanes wide); 1 (< 4 lanes wide)
Path/road condition^4^	0 (path 2 sides poor/road poor); 1 (path good 1 side/road moderate); 3 (path good both sides/road good)
Traffic volume^2^	0 (>14000); 1 (3000–13999); 2 (< 3000 vehicles/day)
Traffic speed^2^	0 (>60 km/h); 2 (60 km/h or less)
Street pattern^1,3^	0 (cul-de-sac); 1(mixed); 2(grid)
Path location from road^4^	0 (no path); 1 (access to a path < 1 m from road); 2 (access to path 1–3 m from road); 4 (access to path >3 m from road)
Alternative routes^4^	0 (no alternative routes); 1 (alternative routes present)
Intersection design^1,3^	0 (3 way (T)); 1 (4 way (+))
Path continuity^4^	0 (not continuous); 1(continuous)
Slope of path/road^4^	0 (access to steep slope only); 1(access to moderate slope only); 2 (access to gentle slope)
Intersection distance^3^	0 (=>250 m); 1 (< 250 m)
Street lights present^4^	0 (no lights); 1 (lights present on one-side of street); 2 (lights present on both sides of street)
Street surveillance^4^	0 (can be seen from < 50% of houses); 1(can be seen from 50–75% of houses); 2 (can be seen from >75% of houses)
Destinations present^4^	0 (no destinations present); 1 (destinations present)
Driveway cross-overs^4^	0 (one per building); 1 (less than one per building or none)

**Aesthetic construct**	
Verge maintenance^4^	0 (< 50% of verges); 1 (50–75% of verges); 2 (>75% of verges)
Garden maintenance^4^	0 (< 50% of houses); 1 (50–75% of houses); 2 (>75% of house)
Cleanliness (rubbish)^4^	0 (lots); 1 (some); 2 (none)
Attractiveness ^4^	0 (not at all); 1(somewhat); 2 (very)
Views (combination)^4^	0 (commercial/no nature); 1 (commercial/nature or urban/commercial); 2 (urban only); 3 (urban/commercial/nature); 4 (urban/nature)
Trees^4^	0 (none); 1 (some/1 side only); 2 (lots/1 side only); 3 (some/2 sides); 4 (some/1 side and lots/other side); 5 (lots/2 sides)
Alikeness of buildings4	0 (all same); 1 (different designs)

For original item wording, category options and scoring refer to Pikora et al. (2003)

^1 ^Segments belong to a certain type of street pattern or intersection design e.g., segment is either part of a 4-way or a 3-way intersection; ^2 ^Sourced from traffic authorities; ^3 ^Derived from GIS and Maps; ^4 ^Derived from SPACES

### Rasch model

The Rasch model[21] is a one-parameter stochastic model that mathematically predicts expected responses to items[22,23]. The residuals between hypothesized and actual response patterns is evidence of the degree of scale unidimensionality[15,24-26]. The Rasch model fit statistics indicate the congruence between the actual and expected pattern of responses across items. In this study the partial credit Rasch model[27,28] was used to examine the fit of the environmental data. This is an extension of the simple Rasch model for dichotomous outcomes[21]. The partial credit model is suitable for items with ordered polytomous outcome scoring categories and allows these categories to vary in number and structure across items[28]. Hence, the operational ordering of the item scoring categories can be examined[29,30]. In the context of this study, the partial credit model estimates the probability that a segment obtains a particular category score on an environmental item as a function of the segment's overall supportiveness for walking. Higher item category scores represented higher supportiveness for walking. It should be noted that multidimensional item response modeling (MIRM) was considered however, only the univariate properties of the constructs were of interest in this study.

### Item exploration and reduction

For each construct (i.e. functional and aesthetic), environmental items were fitted to two separate Rasch models using the exploratory sample: 1) a baseline model including all items believed to belong to the construct; and 2) a revised model, which included items that best represented the construct according to the Rasch model fit statistics. The revised model was cross-validated in the validation sample. Given that no one test of fit is sufficient, the reduction of items was based on the examination of three main statistics: 1) point-biserials; 2) category outcome characteristics; and 3) overall model and variable fit. The Rasch analysis was undertaken using RUMM2020 (RUMM Laboratory Pty Ltd, Murdoch University, Western Australia).

### Evaluation of point-biserial's

Point-biserials (r_pb_) were checked as a preparatory step before examining fit in Rasch analysis, a procedure used to detect initial departure from the expected model[25,31,32]. A negative or low positive point-biserial correlation can indicate that an item is not acting as expected with regard to the underlying construct. Generally, r_pb _> 0.20 are desirable however, items with negative point-biserials or low positive point-biserials (r_pb _< 0.15) were examined further for content. A lower cut-off value was used because of the low number of variables being examined at the beginning of this study. Environmental items that did not appear to be associated with the constructs based on the point-biserials and after reviewing their content were subsequently excluded from the Rasch analysis.

### Evaluation of the scoring model and rescoring

The scoring model represents the category responses or scores for the items[15]. Category Characteristic Curves (CCC) were used to examine the item scoring models. In the context of this study, if an item scoring model is functioning as expected, the probability of obtaining a higher category score on that item would increase as segments overall supportiveness of walking increased. Dysfunctional scoring models can be due to an item not representing the underlying construct or problems either associated with the original scoring categories or how the scoring categories have been collapsed[30,33]. In this study, items that showed dysfunctional scoring were rescored on an individual basis. Based on recently published suggestions for collapsing categories[26,32,34], several rescores of categories were explored before deciding on a final scoring model. The final scoring model for an item required that the rescoring of categories had face validity, improved model fit of the individual item, and where possible reflected a uniform frequency distribution across it's categories.

### Model and item fit statistics

Two types of statistic were used concurrently to provide evidence of variable misfit to the Rasch model: the item-person interaction statistic and the item-trait interaction statistic[30,35]. The item-person interaction statistic (Z_std_) is a standardized residual derived from the difference between the expected or modeled score and the obtained score for each segment to each item[30]. This statistic is determined for each environmental item and can be summarised over the entire set of items.

The item-trait interaction statistic is a chi-square that is determined from the comparison between the expected score and the mean observed score for groups of people (i.e., segments) also known as class intervals, with similar ability (i.e., support for walking) estimates on an item. Five class intervals representing groups of similarly supportive segments were used in the analysis. An item-trait interaction statistic was derived across all environmental items, and if found to be statistically significant (evidence of misfit) then item-trait interaction statistics were investigated at the item level[30].

Measurement models never fully match the data they are intended to represent[36]. Furthermore, the use of large samples leads to even minor levels of misfit being statistically significant when chi-square statistics are used[24,36,37] resulting in rejection of the model, and resulting in the removal of items that are truly related to the underlying construct. Based on these issues, less strict criteria of misfit to the model were used in this study. Items that showed both item-person interactions statistics < -2.5 or >2.5, and statistically significant item-trait interaction statistic (p < 0.01) were removed from further analysis. Because of our large sample size (i.e., n >5000), chi-square statistic was adjusted to a sample size of 500 as this is considered small enough to yield more meaningful interpretation of the chi-square results[11,38,39]. After removal of misfitting items, those remaining were re-entered into the Rasch model and the process repeated until all remaining variables showed sufficient evidence of fit[24]. The final items were then tested in the validation sample to ensure they had acceptable fit. Internal consistency of the constructs were examined using the person separation reliability statistic (r_β_)[40] – a Rasch based version of Cronbach's alpha.

Bivariate correlations between the final functional and aesthetic scales and measures of physical activity were performed using the exploratory dataset. Physical activity data included self-reported fortnightly minutes of recreational walking, transport-related walking, and vigorous-intensity physical activity collected from face-to-face interviews with 1803 SEID 1 respondents [16]. For the correlations, segment data for the functional and aesthetic scales were aggregated to the neighborhood level (i.e., average scale score for segments located within 400 meters of the respondents home).

## Results

### Descriptive statistics

Table 2 presents descriptive statistics for the functional and aesthetic environmental items initially included in the Rasch models. The mean score (i.e., summation of item raw scores) for the functional scale was 20.44 (SD = 3.56 and range = 0 to 35). The initial point-biserials (r_pb_) for the functional environment items suggested that seven variables were less than the predetermined cut-off (r_pb _< 0.15). The summary raw score for the aesthetics scale was 12.34 (SD = 2.79 and range = 0 to 18) and the initial point-biserials for all aesthetic items were > 0.15.

**Table 2:** Means, standard deviations, minima, maxima, and point-biserials for individual environmental indices and environmental scales scores

**Variables**	**Mean**	**SD**	**Min**	**Max**	**r**_**pbis**_^**1**^	**r**_**pbis**_^**2**^
***Functional***	***20.44***	***3.56***	***9***	***30***		
Traffic control devices	0.94	1.08	0	4	.73	.64
Crossing devices	1.86	1.08	0	4	.69	.56
Crossing aids	2.07	0.99	0	4	.70	.56
Road width	0.93	0.25	0	1	.37	.32
Path/road condition	1.03	0.59	0	2	.12	.24
Traffic volume	1.67	0.63	0	2	.53	.42
Traffic speed	0.99	0.12	0	1	.19	
Street pattern	1.43	0.79	0	2	.17	.18
Alternative routes	0.30	1.77	0	1	.20	.31
Intersection design	0.19	0.39	0	1	.15	.19
Path continuity	0.73	0.44	0	1	.14	.21
Slope of path/road	1.68	0.56	0	2	.12	.22
Intersection distance	0.19	0.39	0	1	.10	
Street lights present	1.07	0.40	0	2	-.12	
Street surveillance	1.66	0.62	0	2	.27	.27
Destinations present	0.35	0.48	0	1	.11	.27
Path location	1.57	1.27	0	3	.18	.15
Driveway crossovers	0.09	0.29	0	1	-.10	
***Aesthetics***	***12.34***	***2.79***	***1***	***18***		
Verge maintenance	1.51	0.68	0	2	.50	.63
Garden maintenance	1.64	0.63	0	2	.50	.62
Cleanliness	1.79	0.45	0	2	.43	.52
Attractiveness	1.01	0.47	0	2	.49	.51
Views	2.50	1.08	0	4	.40	.39
Trees	3.54	1.79	0	5	.68	.59
Alikeness of buildings	0.34	0.47	0	1	.16	.20

^1 ^Initial point-biserial correlation

^2^Point-biserial correlation after category rescoring and variable reduction (r_pbis _< 0.15)

### Model fit

The results of the Rasch analyses are presented in Table 3. For both the functional and aesthetic environment scales, the fit of the original model was unacceptable, as shown by the high standardized fit residuals (M = -3.10 and M = 2.87, respectively) and statistically significant chi-squares (p < 0.001).

**Table 3:** Functional environment and aesthetic scale fit to the Rasch model

		**Variable-trait interaction Z**_**std**_		**Variable-trait interaction χ**^**2 **^**statistic**		
**Construct**	**N items**	**Mean**	**SD**	**df**	**χ**^**2**^	**p- value**

**Functional environment**						
Initial model	18	-3.10	13.47	72	417.23	< .001
Revised model	12	0.27	2.52	48	65.22	0.049
Cross-validation model	12	0.36	2.65	48	53.29	0.278
						
**Aesthetic environment**						
Initial model	7	2.87	6.86	21	114.02	< .001
Revised model	4	0.60	2.55	12	15.06	0.238
Cross-validation model	4	0.74	3.42	12	18.66	0.097

Zstd = standardized residual; Statistical significance considered at p < 0.01

**Figure 1:**
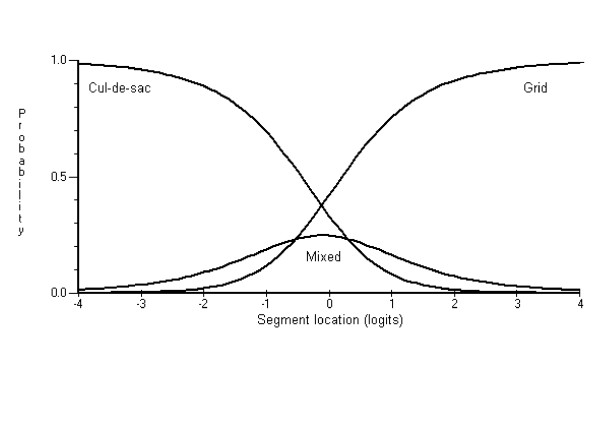
Category Characteristic Curve showing dysfunctional scoring model for street pattern.

**Figure 2:**
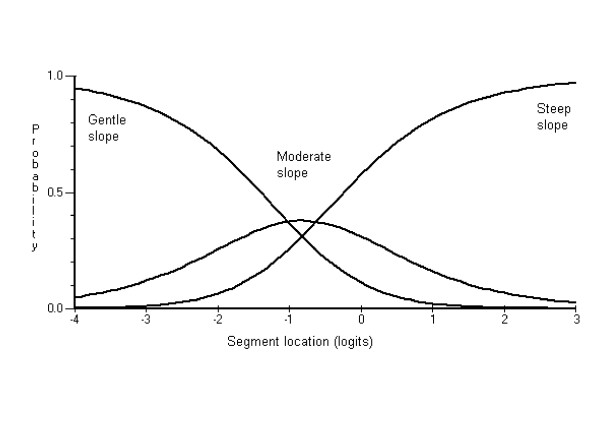
Category Characteristic Curve showing normal functioning scoring model for path/road slope.

The scoring model was first examined to determine which item scoring categories were not functioning as expected. Evaluating the CCCs served to refine the scoring system by identifying item categories that might be collapsed. Examination of the CCCs indicated that seven items from the functional environment scale (*street pattern, path location*, *surveillance, traffic volume, traffic control devices, crossing devices *and *crossing aids*) and three from the aesthetics environment scale (*verge trees, views, and cleanliness*) showed dysfunctioning score categories. These items were subsequently rescored. The CCCs in Figures 1 and 2 respectively, show examples of items with dysfunctioning and normal functioning scoring categories. Note the middle CCC score category (i.e., mixed street pattern) in Figure 1 never has a higher probability of being selected compared with the other two categories.

Items with point-biserials < 0.15 were eliminated as they discriminated poorly and made a limited contribution to the scale score. After category rescoring, the point-biserials of some items differed from the initial values presented in Table 2. For clarity, point-biserials of the items remaining following rescoring and variable reduction (based on the r_pb _< 0.15 and content evaluation) are presented in the final column of Table 2. These latter values were used in the next step of the analysis. Four items were eliminated from the functional environment scale (*driveway crossovers*, *street lights present, traffic speed*, and *intersection distance*).

Due to high standardized residuals (i.e., -2.5 > Z_std _> 2.5) and significant variable-trait chi-square values (p < 0.01), *traffic control devices *(Z_std _=-15.93; chi-square = 28.37, df = 4, p < 0.01)*and crossing devices *(Z_std _= -12.24; chi-square = 21.34, df = 4, p < 0.01) were removed from the functional environment scale and *garden maintenance *(Z_std _= -10.85; chi-square = 24.55, df = 3, p < 0.01), *alikeness of design *(Z_std _= 17.09; chi-square = 39.72, df = 3, p < 0.01), and *attractiveness *(Z_std _= -6.69; chi-square = 11.86, df = 3, p < 0.01) were removed from the aesthetic environment scale. Following the removal of these items, the remaining items in each scale showed evidence of model fit (chi-square statistics p > 0.01).

The Rasch analysis of the shortened functional environment scale indicated an adequate fit as shown by the standardized fit residuals (model M = 0.27, SD = 2.52) and the chi-square statistic (p < 0.049). To cross-validate the results and assess the generalizability of the findings, the Rasch analyses were replicated using the data from the validation sample. Results of the cross-validation showed that the data adequately fitted the revised model (see Tables 3 and 4). Furthermore, based on data from the exploratory dataset, the Pearson correlation between the functional and aesthetic scales was r = 0.11 (p < 0.001). The final set of items for the functional and aesthetic environment scales and location and model fit information are shown in Table 4 and their category response score structures (i.e., following rescoring) are presented in Table 5.

**Table 4:** Final variable locations, variable-trait standardized residuals, and variable-trait chi-square statistics for the training and cross-validation samples

**Variables**	**Loc.**	**Z**_**std**_	**χ**^**2**^	**Sig.**	**Loc.**^**a**^	**Z**_**std**_^**a**^	**χ**^**2a**^	**Sig.**^**a**^
**Functional**								
Crossing aids	-0.04	2.09	9.54	.049	-0.01	0.70	7.12	.130
Road width	-2.11	-1.88	1.65	.799	-2.15	-1.55	1.71	.788
Path/road condition	0.48	-2.90^b^	2.05	.726	0.47	-3.04^b^	0.71	.950
Traffic volume	-0.50	3.73^b^	7.26	.123	-0.45	4.59^b^	7.28	.122
Street pattern	-1.03	-3.48^b^	9.82	.044	-1.07	-3.52^b^	7.98	.092
Path location from road	0.35	1.78	7.63	.106	0.30	0.77	7.97	.093
Alternative routes	1.40	0.76	1.83	.767	1.46	1.66	2.03	.731
Intersection design	2.09	-0.93	3.08	.544	2.11	-1.92	3.24	.517
Path continuity	-0.53	-1.21	7.02	.135	-0.57	0.01	4.34	.362
Slope of path/road	-0.82	0.78	6.72	.152	-0.81	0.86	4.53	.338
Street surveillance	-0.48	4.65^b^	4.69	.320	-0.39	4.98^b^	2.94	.568
Destinations present	1.18	-0.11	3.91	.418	1.10	0.81	3.43	.488
								
**Aesthetics**								
Verge maintenance	1.19	-0.62	0.92	.762	1.23	-2.07	3.56	.313
Cleanliness	-2.11	-2.40	6.73	.059	-2.08	-2.28	8.74	.033
Views	-0.27	3.03^b^	4.13	.238	-0.27	4.40^b^	2.16	.472
Trees	1.19	2.38	1.57	.523	1.12	2.92^b^	3.84	.279

Note: Loc. = location in logits; Z_std _= standardized residual; Statistical significance considered at p < 0.01; ^a ^= Cross-validation sample ^b ^= Z_std _< -2.5 or >2.5

**Table 5:** Final category response score structures for the final set of environmental variables

	**Category**						
	**Rescored**	**1**	**2**	**3**	**4**	**5**	**6**

**Functional variables**							
Crossing aids	Yes	0	1	1	2	2	
Road width	No	0	1				
Path/road condition	No	0	1	2			
Traffic volume	Yes	0	0	1			
Street pattern	Yes	0	1	1			
Path location from road	Yes	0	0	1	1		
Alternative routes	No	0	1				
Intersection design	No	0	1				
Path continuity	No	0	1				
Slope of path/road	No	0	1	2			
Street surveillance	Yes	0	0	1			
Destinations present	No	0	1				
							
**Aesthetics variables**							
Verge maintenance	No	0	1	2			
Cleanliness	Yes	0	1	1			
Views	Yes	0	1	2	2	2	
Trees	Yes	0	1	1	1	2	2

For category response score descriptions refer to Table 1.

### Scale representation

**Figure 3:**
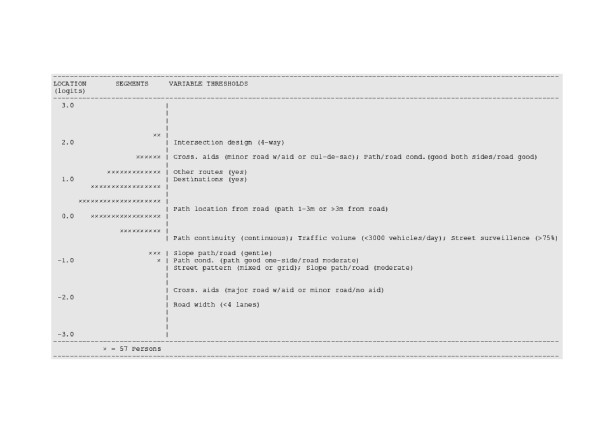
Segment and item threshold location on the functional environment scale.

**Figure 4:**
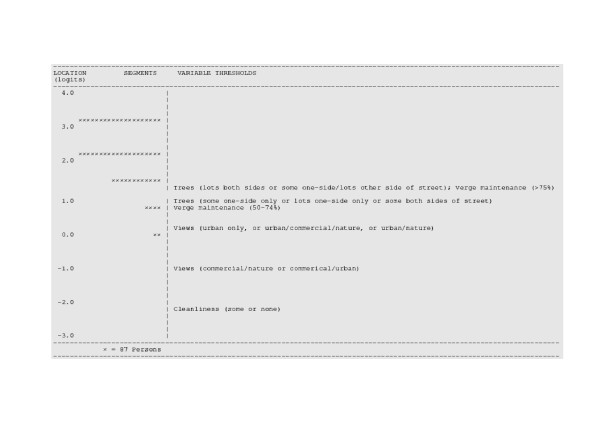
Segment and item threshold locations on the aesthetic environment scale.

Figures 3 and 4 show the distribution of segment and item threshold locations along the same continuum for the functional and aesthetic environment scales, respectively. Both segments and items have a common measurement unit referred to as a logit (i.e., log odds unit), which allows their locations on the item-segment map to be compared. Segments located below item thresholds are less likely to have the attribute to which the thresholds pertain, and segments above item thresholds are more likely to have the attributes to which the thresholds pertain. The mean segment location for the functional environment scales was 0.60 (SD = 0.72); while for the aesthetic scale, mean segment location was 2.24 (SD = 0.95). The range of functional item threshold locations (-2.11 to 2.09 logits) overlapped with most of the segment locations. The second category threshold for crossing aids (1.64 logits) and path/road condition (1.79 logits) was higher than all other thresholds except for that of intersection design. The second category thresholds for slope of path/road (-0.63 logits) was located lower than the thresholds for path continuity, traffic volume, and street surveillance. Highly supportive segments that belonged to a 4-way intersection were also more likely to have all other supportive functional environmental attributes (i.e., all environmental attributes in lower locations). Most segments had road widths less than four lanes and crossing aid/road type combinations that were more supportive of walking than having a major road with an aid or a minor road without an aid (Figure 3).

Segments with more than 75% of verges maintained (1.40 logits) were also more likely to have all other supportive aesthetic environmental attributes (Figure 4). Segments with either lots of trees on both sides of the street or some trees on one-side with lots on the other (1.36 logits) were more likely to have 50–74% of verges maintained, either urban only, urban and commercial and nature, or urban and nature views, and some or no street rubbish. The majority of segments (~ 88%) were located above the highest item threshold (i.e., 1.40 logits) indicating a ceiling effect for the aesthetic environment items in this sample of segments. The possible mismatch between the segment and item distributions influences the segment separation indices. Segment separation indices for the functional environment and aesthetic environment scales were considered low (r_β _= 0.19 and 0.35, respectively). However, the segment separation indices of the original set of items were also low to moderate (functional scale r_β _= 0.35, and aesthetic scale r_β _= 0.51).

Low or non-existent bivariate correlations between the neighborhood level functionality score and fortnightly minutes of recreational walking (r = 0.00, p = 0.86), transport-related walking (r = 0.07, p = 0.003), and vigorous-intensity physical activity (r = 0.01, p = 0.80) were found. Similarly, low or non-existent associations were found between the neighborhood level aesthetics score and recreational walking (r = 0.01, p = 0.66), transport-related walking (r = -0.07, p = 0.003), and vigorous-intensity physical activity (r = 0.06, p = 0.02).

## Discussion

This study explored how environmental attributes supportive of walking are related and described a process of deriving environmental indices, using the Rasch model. Given the vast number of attributes found in the built environment, a valid means of developing summary indices is essential. The development of environmental indices is supported by the fact that environmental attributes exert their affects on behavior collectively and not necessarily in isolation[7,41].

Scale reliability

Items from the SPACES instrument have been shown to have acceptable test-retest reliability [8]. However, the scales developed in this secondary analysis from the SPACES items and Geographical Information Systems data, had less than desirable internal consistency (i.e., low separation statistics). For the functional and aesthetic scales, the low number of items, the attenuated range and variability of segment scores, and the lack of overlap between the level of supportiveness of the items and level of supportiveness of the segments (i.e., the segment separation reliability decreases as the mismatch becomes more pronounced) may have contributed to lower segment separation statistics[40].

A source of low variability might be the item scoring models or the items themselves not being sensitive enough to detect differences among the segments. In particular, the lack of overlap between segments and aesthetic items (see Figure 4) suggests that additional items are needed to differentiate among the majority of segments. More subtle aesthetic qualities may need to be captured in order to differentiate among similar segments in this study. Items capturing attributes relating to architecture, house design, the color of buildings, and attractiveness of gardens may increase the variability in the aesthetics score among segments. A caveat of including this level of detail is that from a policy perspective it might not be possible to intervene on such subtle attributes. For example, characteristics of residential garden landscaping are largely the responsibility of home owners, although incentives could be introduced to encourage owners to maintain them. Hence, measuring such specific features will be of little relevance for encouraging change in physical activity behavior if modifying some environmental features is difficult to implement.

The homogeneity of segment attributes – reflecting the way in which segments were sampled in SEID 2 (i.e., all segments audited within 400 meters of the respondent's home) – likely reduced the variability in these data. *Post hoc *examination of intra-cluster correlations (ICC) for the two scales suggests that segments within neighborhoods were somewhat homogenous (i.e., functional ICC = 0.27 and aesthetic ICC = 0.28). To some degree, similarity among segments and neighborhoods is unavoidable given that often at a state, regional, city and neighborhood level over-arching laws and legislation such as zoning and design legislation govern what can or cannot be built. Furthermore, environmental attributes measured at the segment level may actually reflect street or neighborhood level characteristics (e.g., street speed limits, street design) resulting in adjacent segments sharing the same attributes. Moudon and Lee [7] propose that objective measures of physical environments need to have sufficient variability. Therefore, study designs which maximize environmental variability need to be implemented. For example, the SMARTRAQ study in Oregon, is selectively sampling segments within neighborhoods (i.e., one segment on a main road, one on a service road), rather than collecting data on all segments within a neighborhood [4].

A limitation of using Rasch analysis on the existing dataset is that the number of items had already been reduced in the initial item selection process. The low number of variables, and the subsequent attenuated range in available scale scores, from the beginning was a limitation of this study. Rasch analysis may therefore be more effectively used during the item development phase, than after the final variables have already been decided upon[11,26]. In situations where there are a large number of items, results obtained from Rasch analysis may serve to detect items that need rewording or need more sensitive category scoring models[11,26]. For example, in the current study, the fact that *garden maintenance *did not fit the Rasch model may have been due to the subjectiveness of the item term "maintenance" and the response options offered (i.e., < 50%, 50% to 75%, >75% gardens in a segment are maintained). Others have found that item subjectivity contributes to low reliability of environmental audits[42]. To maintain brevity, the present paper did not explore reasons why some item scoring models did function as expected. Nevertheless, we acknowledge that this should be considered, particularly in the development and piloting stage of an instrument.

### Scale validity

Items that show evidence of sufficient fit to the Rasch model are considered to contribute to a single underlying construct[15,24-26]. The locations of environmental attributes or their hierarchy represent their supportiveness for walking in relation to other attributes. Highly supportive segments that were part of a 4-way intersection were also more likely to have all other supportive environmental attributes (see Figure 3). The presence of 4-way intersections in a neighborhood may contribute to greater neighborhood connectivity (i.e., increased route directness)[41,43,44]. Similarly, direct routes to destinations (e.g., from home to the shop) are supportive of walking because they encourage individuals to walk for transportation[41,43,44]. Our results suggest that having 4-way intersections may be even more supportive for walking because they are associated with having other supportive environmental attributes. Segments of moderate supportiveness were more likely to have all other supportive attributes except destinations, other routes, and being part of a 4-way intersection. Segments less supportive of walking were more likely to have continuous paths, low traffic volumes, good street surveillance, gentle sloping paths/roads, belonged to a mixed or grid street pattern, and have less than 4-lanes, but were less likely to have the other supportive environmental attributes, found in moderate and high supportive segments.

For aesthetic attributes, segments with trees and maintained verges were more likely to have appealing views and higher cleanliness. However, the majority of the segments were located on the high aesthetic side on this continuum's attribute (i.e., above the location of trees and verge maintenance) suggesting that the scale needs more items or variables which discriminate along the full aesthetic continuum and, in particular, at the higher end. It might be necessary to develop or include items which capture aesthetic attributes that demand higher levels of endorsement than those currently included in the SPACES instrument. In addition, it might be necessary to make the item scoring categories more sensitive to minor attribute differences so that current items can discriminate among high supportive segments (e.g., increasing the available range of item and scale scores).

The correlations between the functional and aesthetic scales and the physical activity behaviors provide weak support for the predictive validity of these scales. Neither the functional nor the aesthetics scale was associated with recreational walking; however, both scales were associated with transport-related walking. The functional scale was positively associated with minutes of transport-related walking while the aesthetics scale was negatively associated with transport-related walking. The latter finding is not entirely unexpected given that mixed associations have been found between aesthetics and walking [1-3]. The aesthetics scale was also positively associated with minutes of vigorous-intensity physical activity. The weak correlations between the environmental scales and physical activity in this study may have resulted from the method used to derive these scales. Using the same data examined in this study, Pikora et al. [6] found a positive association between functionality and recreational walking in the neighborhood however, no association was found between functionality and transport-related walking in the neighborhood or aesthetics and any walking behavior. The different types of walking behavior examined in each study (i.e., neighborhood walking versus non-context specific walking) may explain the difference in results. Furthermore, the different methods for aggregating environmental variables into overall indices of functionality and aesthetics in Pikora et al.'s [6] and the present study may also explain the lack of correspondence. For example, Pikora et al. [6] aggregated variables according to the original conceptual framework, which also included weighting attributes according to their importance for encouraging walking [10]. In contrast the present study did not use any weighting, and the scales were derived empirically using Rasch analysis. While the lack of variability in the SEID 2 data is considered a limitation and likely cannot be overcome regardless of which method is used to derive the environmental scales [6], these results together suggest that different methods of scale development, even when using the same data, can lead to different conclusions.

Given the complicated interrelationships among physical environmental attributes [41], empirical as well as conceptual evidence should be used to derive these scales. Exploratory approaches often empirically examine the data structure before assigning meaning to the constructs. In this study the approach taken was mainly empirically driven however, our analysis began with a theoretical model derived from previous research [10]. Rasch analysis was used to confirm this theoretical model. An exploratory approach was then taken to obtain better fit of data to the model. Research investigating measurement of the built environment is in its infancy. Hence, specifying constructs prior to examining the data structure may have restricted the findings of our study. For example, the inclusion of other environmental constructs may better represent these data. The empirically-driven approach taken following the failure of the items to initially fit the theoretical constructs, means that replication of our findings in other built environments is not guaranteed. However, testing the final items in the validation sample is a strong point of this study, and suggests replication of our findings among segments with similar environmental characteristics. Further research which investigates the conceptual and operational definitions of objective environmental constructs and attributes is needed.

## Conclusion

Given the complicated interrelationships among physical environmental attributes, empirical as well as conceptual evidence should be used to form these scales. However, it is important that scales representing the supportiveness of the environment are both valid and reliable. Although the functional and aesthetic scales derived in this study showed inconsistencies in their statistical properties the study provides information about the process of constructing environmental scales from several sources. The Rasch model is dependent on a replicable pattern of endorsement across items hence, misfiting items and the low separation statistics might indicate that there is no logical relationship between certain environmental attributes. More examples of procedures for measuring the built environment and techniques for analyzing environmental data are needed to guide future research in this area.

## Competing interests

The author(s) declare that they have no competing interests.

## Authors' contributions

GRM, LCM, and BG-C conceived this study. GRM and LM conducted the analysis, and drafted the manuscript. All authors commented on draft manuscripts.
